# A Comparison of Lung Ultrasound and Computed Tomography in the Diagnosis of Patients with COVID-19: A Systematic Review and Meta-Analysis

**DOI:** 10.3390/diagnostics11081351

**Published:** 2021-07-27

**Authors:** Mengshu Wang, Xufei Luo, Ling Wang, Janne Estill, Meng Lv, Ying Zhu, Qi Wang, Xiaojuan Xiao, Yang Song, Myeong Soo Lee, Hyeong Sik Ahn, Junqiang Lei, Jinhui Tian

**Affiliations:** 1 Evidence-Based Medicine Centre, School of Basic Medical Sciences, Lanzhou University, Lanzhou 730000, China; wangmsh19@lzu.edu.cn (M.W.); ying.zhu189@gmail.com (Y.Z.); 2 Department of Radiology, The First Hospital of Lanzhou University, Lanzhou 730000, China; leijq1990@163.com; 3 School of Public Health, Lanzhou University, Lanzhou 730000, China; luoxf2016@163.com; 4 Department of Ultrasound, People’s Hospital of Gansu Province, Lanzhou 730000, China; gssrmyywl2016@163.com; 5 Institute of Global Health, University of Geneva, 1211 Geneva, Switzerland; janne.estill@stat.unibe.ch; 6 Department of Nephrology, Children’s Hospital of Chongqing Medical University, Chongqing 400014, China; lvm2016@163.com; 7 National Clinical Research Center for Child Health and Disorders, Ministry of Education Key Laboratory of Child Development and Disorders, China International Science and Technology Cooperation Base of Child Development and Critical Disorders, Chongqing 400014, China; 8 Chongqing Key Laboratory of Pediatrics, Chongqing 400014, China; 9 Department of Health Research Methods, Evidence and Impact, Faculty of Health Sciences, McMaster University, Hamilton, ON L8S 4L8, Canada; wangq87@mcmaster.ca; 10 McMaster Health Forum, McMaster University, Hamilton, ON L8S 4L8, Canada; 11 Department of Radiology, The Eighth Affiliated Hospital, Sun Yat-sen University, Shenzhen 518033, China; xiaoxj90@126.com; 12 Iberoamerican Cochrane Centre-Biomedical Research Institute Sant Pau (IIB Sant Pau), 08025 Barcelona, Spain; yangsongcochrane@gmail.com; 13 Korea Institute of Oriental Medicine, Daejeon 34054, Korea; drmslee@gmail.com; 14 Korean Convergence Medicine, University of Science and Technology, Daejeon 34113, Korea; 15 Tianjin University of Traditional Chinese Medicine, Tianjin 301617, China; 16 Department of Preventive Medicine, Korea University, Seoul 02841, Korea; ahnhann@gmail.com; 17 Korea Cochrane Centre, Seoul 02841, Korea; 18 Institute for Evidence Based Medicine, College of Medicine, Korea University, Seoul 02841, Korea; 19 Department of Preventive Medicine, College of Medicine, Korea University, Seoul 02841, Korea; 20 Intelligent Imaging Medical Engineering Research Center of Gansu Province, Lanzhou 730000, China; 21 Accurate Image Collaborative Innovation International Science and Technology Cooperation Base of Gansu Province, Lanzhou 730000, China; 22 Key Laboratory of Evidence-Based Medicine and Knowledge Translation of Gansu Province, Lanzhou University, Lanzhou 730000, China

**Keywords:** COVID-19, systematic review, POCUS, lung ultrasound, computed tomography

## Abstract

Background Lung ultrasound (LUS) and computed tomography (CT) can both be used for diagnosis of interstitial pneumonia caused by coronavirus disease 2019 (COVID-19), but the agreement between LUS and CT is unknown. Purpose to compare the agreement of LUS and CT in the diagnosis of interstitial pneumonia caused by COVID-19. Materials and Methods We searched PubMed, Cochrane library, Embase, Chinese Biomedicine Literature, and WHO COVID-19 databases to identify studies that compared LUS with CT in the diagnosis of interstitial pneumonia caused by COVID-19. We calculated the pooled overall, positive and negative percent agreements, diagnostic odds ratio (DOR) and the area under the standard receiver operating curve (SROC) for LUS in the diagnosis of COVID-19 compared with CT. Results We identified 1896 records, of which nine studies involving 531 patients were finally included. The pooled overall, positive and negative percentage agreements of LUS for the diagnosis of interstitial pneumonia caused by COVID-19 compared with CT were 81% (95% confidence interval [CI] 43–99%), 96% (95% CI, 80–99%, I^2^ = 92.15%) and 80% (95%CI, 60–92%, I^2^ = 92.85%), respectively. DOR was 37.41 (95% CI, 9.43–148.49, I^2^ = 63.9%), and the area under the SROC curve was 0.94 (95% CI, 0.92–0.96). The quality of evidence for both specificity and sensitivity was low because of heterogeneity and risk of bias. Conclusion The level of diagnostic agreement between LUS and CT in the diagnosis of interstitial pneumonia caused by COVID-19 is high. LUS can be therefore considered as an equally accurate alternative for CT in situations where molecular tests are not available.

## 1. Background

During the first year of the pandemic, Coronavirus Disease 2019 (COVID-19) has caused substantial harm in all aspects of life and a great loss of human, material, and financial resources. Diagnosis is a critical step for the treatment and prognosis of COVID-19. Currently, the reverse-transcriptase polymerase chain reaction (RT-PCR) for viral load is considered as the gold standard in the diagnosis of COVID-19 [1]. However, receiving the results of RT-PCR can take at least several hours, and in some circumstances, particularly in emergency situations, more rapid diagnostic methods are needed. Other common diagnostic methods for COVID-19 include chest computed tomography (CT), X-ray. Each of these methods has advantages in some aspects in the detection of COVID-19. However, one single study has shown that all of them have a low specificity compared with RT-PCR in diagnosing COVID-19 [2]. The use of imaging for patients with acute or severe COVID-19 is also inconvenient for the patients.

Lung ultrasound (LUS) refers to the application of ultrasound technology in the diagnosis and screening of respiratory diseases. LUS can be used to evaluate pleural abnormalities, to guide thoracentesis and related procedures, and to improve the accuracy and safety of identifying pleural disease and performing transpleural access-related procedures [3]. Compared to traditional pleural radiography, LUS has a multitude of advantages: it is radiation-free, uses portable equipment, the imaging is real-time and can be displayed dynamically [4]. Point-of-care ultrasonography (POCUS), a LUS tool widely used in emergency and critical care departments, is particularly practical as it can be used at the patient’s bedside, saving time and capacity. LUS and CT can both be used for diagnosis, assessment of disease severity, and evaluation of prognosis of interstitial pneumonia caused by COVID-19. In this systematic review, we aim to comprehensively identify the diagnostic agreement between LUS and CT in the diagnosis of interstitial pneumonia caused by COVID-19. We present this article in accordance with the PRISMA-DTA statement [5].

## 2. Methods

We have registered this systematic review at OSF REGISTRIES and the registration DOI is 10.17605/OSF.IO/ZY7FU.

### 2.1. Inclusion and Exclusion Criteria

#### 2.1.1. Inclusion Criteria

We limited the search to studies that compared LUS with CT in the diagnosis of interstitial pneumonia caused by COVID-19. We included peer-reviewed papers published in English or Chinese that met the following conditions: (1) participants were patients with confirmed or suspected COVID-19 or asymptomatic SARS-CoV-2 infection; (2) the diagnostic method was ultrasound diagnosis (including POCUS) in the intervention group, and CT in the control group; and (3) a two-by-two contingency table comparing the diagnosis results with LUS and CT could be calculated.

#### 2.1.2. Exclusion Criteria

We excluded narrative reviews, letters, and conference abstracts; duplicate publications; studies with insufficient data; and studies from which data could not be extracted.

### 2.2. Search Strategy

We systematically searched Medline, Embase, The Cochrane Library, Chinese Biomedicine Literature, and WHO COVID-19 database from 1 January 2020 to 15 January 2021 to identify studies on the use of LUS in the diagnosis of COVID-19. We used the following search strategy, adapted for the requirements of each database if necessary: (“COVID-19” OR “2019-nCov” OR “SARS-CoV-2”) AND (“ultrasonography” OR “ultrasound” OR “echography” OR “ultrasonics” OR “ultrasonic diagnosis” OR “ultrasonic echo” OR “ultrasonic examination” OR “ultrasonic scanning”). We also searched the reference lists of the identified articles to find additional studies. The details of the search strategy are shown in Supplementary Material 1.

### 2.3. Article Selection and Data Extraction

Two reviewers first screened all titles and abstracts, and then the full texts of articles deemed potentially eligible, independently according to the inclusion and exclusion criteria. Disagreements were solved by consensus or consultation with a third reviewer. The following information was extracted: (1) basic information (the first author, publication date, country or region of participants, and sample size), (2) patient information (age, gender, enrollment time of patients, setting where the study was conducted), and (3) the values of the two-by-two contingency table comparing the diagnostic outcomes with LUS and CT. If the contingency table could not be extracted from the article, we contacted the corresponding author for the information. If we could not retrieve the necessary data despite contacting the authors, the article was excluded.

### 2.4. Assessment of Risk of Bias and Quality of Evidence

Two investigators used the Quality Assessment of Diagnostic Accuracy Studies 2 (QUADAS-2) tool to assess the risk of bias in the included studies independently [6]. The QUADAS-2 tool covers both risk of bias and applicability. The risk of bias section consists of four domains (patient selection, index test, reference standard, and flow and timing) and the applicability section of three domains (patient selection, index test, and reference standard). The risk of bias or concerns in applicability in each domain are rated as either “low”, “high”, or “unclear”. Review Manager (RevMan) Version 5.4. (The Cochrane Collaboration, 2020) was used to present the results. In case of disagreement consensus was reached by discussion or consultation with a third researcher.

The quality of evidence was evaluated using the GRADE approach. For outcomes on diagnostic accuracy, the assessment starts by assuming high quality of evidence, which is then downgraded according to the risk of bias, indirectness, inconsistency, impreciseness, and publication bias, and upgraded for dose-response effect, large residual effects, and lack of bias and confounding [7].

### 2.5. Statistical Analysis

We calculated the pooled overall percent agreement, positive percent agreement, and negative percent agreement comparing LUS in the diagnosis of COVID-19 with CT as the reference. The overall percent agreement is the proportion of all test results that were in agreement with the two methods. The positive and negative percent agreements are equivalent to sensitivity and specificity, respectively, in situations where the reference is not necessarily the gold standard. The I^2^ statistics and Q test were used to measure and interpret the heterogeneity. Meta-analysis was performed after we had confirmed that there was no statistical heterogeneity. We used either the fixed or randomized effect model according to the guidance in the Cochrane Handbook for Systematic Reviews of Diagnostic Test Accuracy. We also calculated the diagnostic odds ratio (DOR) and area under the summary receiver operating characteristic (SROC) curve assuming CT as the reference standard. The analyses were performed in STATA 14 (Stata/MP 14.0 for Mac (64-bit Intel), Revision 22 April 2015, Copyright 1985–2015 StataCorp LP) software. The significance level of the meta-analysis was set at α = 0.05.

## 3. Results

### 3.1. Literature Selection Process

**Figure 1:**
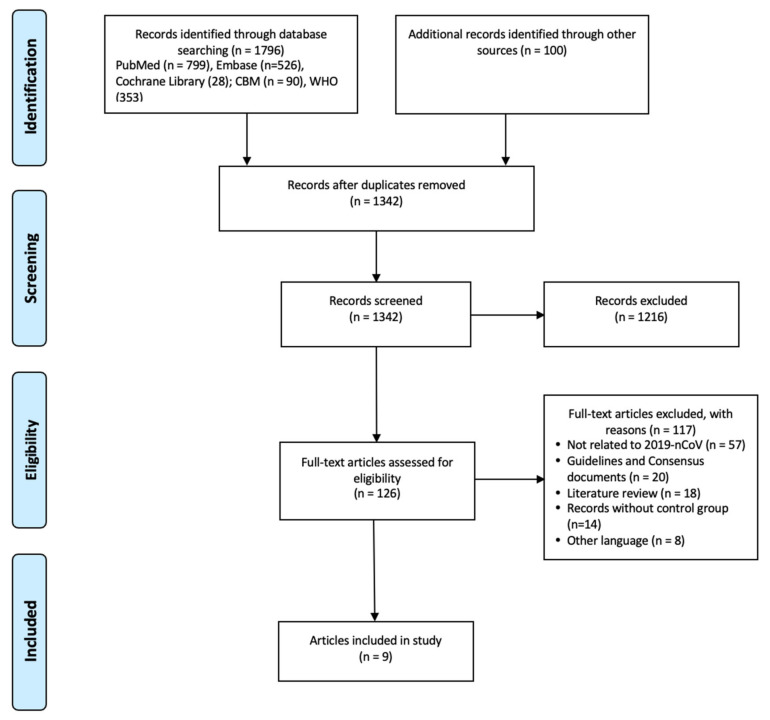
Flowchart of the literature search and screen.

A total of 1896 documents were retrieved. After reading the titles, abstracts, and full text of the documents, nine articles were included in the analysis [8,9,10,11,12,13,14,15,16] (Figure 1).

### 3.2. Characteristics of the Included Studies

A total of nine studies involving 531 patients were included, of which, three studies were from Turkey, and one each from the United States, Germany, Spain, Belgium, Italy, and China (Table 1). The study subjects included in the study were recruited between February and May of 2020 with sample sizes ranging from nine to 131. Four studies contained patients suspected for COVID-19, four contained patients with confirmed case of COVID-19, and one contained asymptomatic patients suspected to be infected with SARS-CoV-2. Only one study reported that they used high-resolution CT.

### 3.3. Risk of Bias in the Included Studies

**Figure 2:**
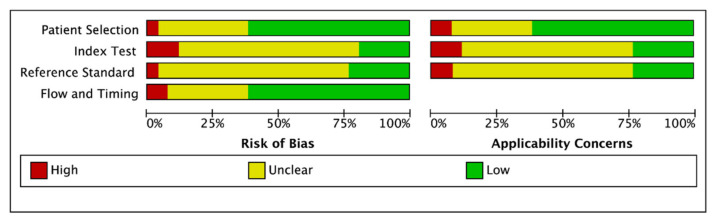
The risk of bias in the included studies.

The results of risk of bias in the included studies are shown in detail in Figure 2. Four studies had a high or unclear risk of bias in patient selection, four studies in flow and timing, seven studies in reference standard, and seven studies in index test.

### 3.4. Agreement of LUS for the Diagnosis of COVID-19

**Figure 3:**
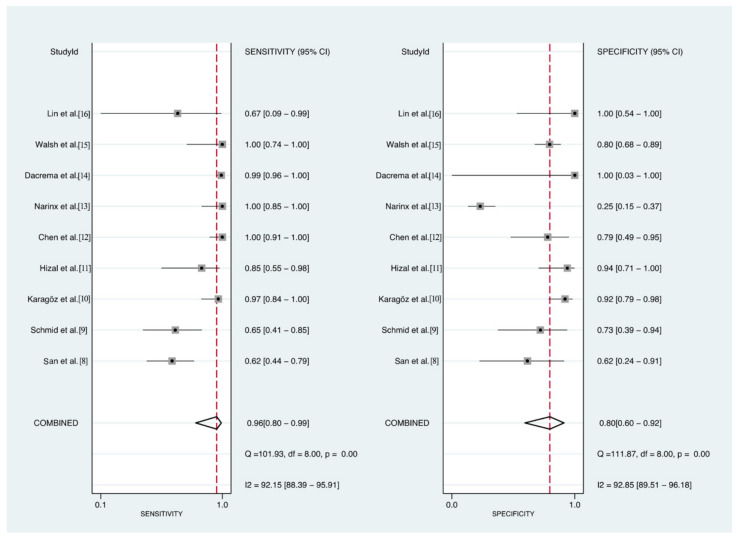
Positive (**left** panel) and negative (**right** panel) percent agreement of lung ultrasound for the diagnosis of interstitial pneumonia caused by COVID-19.

**Figure 4:**
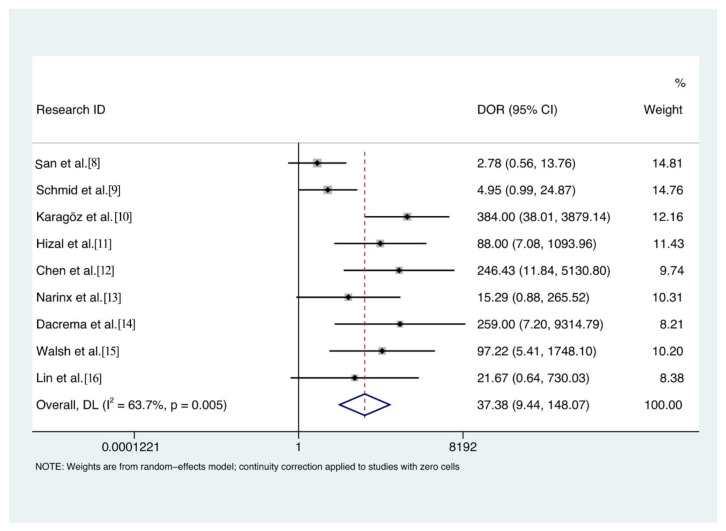
Diagnostic odds ratio of lung ultrasound for the diagnosis of interstitial pneumonia caused by COVID-19.

**Figure 5:**
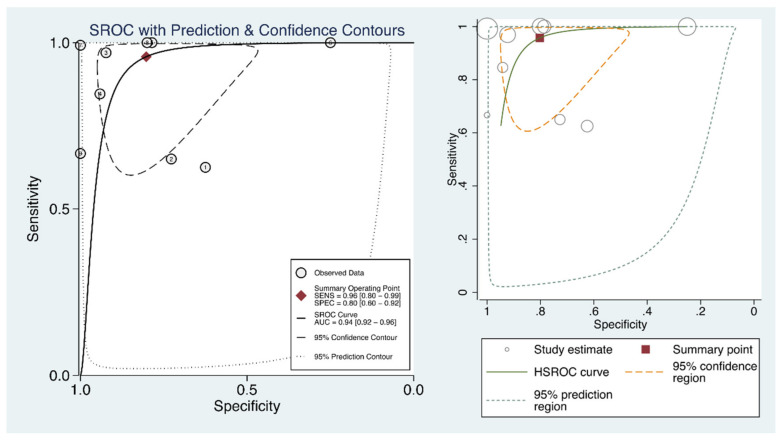
The summary receiver operating characteristic curve of lung ultrasound for the diagnosis of interstitial pneumonia caused by COVID-19.

The pooled overall percent agreement was 81% (95% confidence interval [CI], 43–99%), positive percent agreement 96% (95% CI, 80–99%, I^2^ = 92.15%), and negative percent agreement 80% (95%CI, 60–92%, I^2^ = 92.85%) (Figure 3). The DOR was 37.4 (95% CI, 9.4–148.5, I^2^ = 63.9%) (Figure 4), and the area under the SROC curve 0.94 (95% CI, 0.92–0.96) (Figure 5). The quality of evidence for both positive and negative percent agreement was low because of heterogeneity and risk of bias.

## 4. Discussion

### 4.1. Principal Findings

Our study identified nine studies comparing LUS with CT for the diagnosis of interstitial pneumonia caused by COVID-19 [8,9,10,11,12,13,14,15,16]. In four out of five cases the diagnoses done by LUS and CT were in agreement. In particular, LUS was able to detect COVID-19 in 96% of patients who were diagnosed positive with CT. The quality of evidence was however low. LUS and CT therefore have comparable reliability in diagnosing COVID-19 in patients at emergency departments.

Ultrasound can yield high resolution images of anatomical structures quickly and in a timely manner. It can also be applied to the examination of lungs, stomach, and other chest structures and to rapidly diagnosis or confirmation of the cause of hemodynamic instability [17]. As early as during the pandemic of influenza A in 2003, LUS was shown to yield results comparable with chest imaging tools for the early diagnosis of H1N1 at the emergency department.

In diagnosing pulmonary diseases or disorders, such as pneumothorax, pleural effusion, pneumonia, chronic obstructive pulmonary disease/asthma, pulmonary edema, and acute respiratory distress syndrome (ARDS), the sensitivity and specificity of LUS have been shown to be higher than those of CT [18]. In patients with ARDS, the capabilities of chest X-ray and LUS in identifying patients at high risk of death have been shown to be equivalent [19]. LUS has been commonly used in the diagnosis of acute respiratory failure as a basic tool that can help improve the diagnosis in intensive care setting. When combined with standard diagnostic methods, LUS can expedite the management of emergency care.

POCUS is one of the more common types of ultrasound, a compact equipment that can be used at the patient’s bedside for diagnostic or adjunctive confirmation of diseases [20]. POCUS is currently attracting a lot of attention in intensive care units (ICU) due to its many advantages. POCUS allows to delay or even avoid the need of transfer to the radiology department, and prevent exposure to radiation [21]. POCUS can also guide life-saving therapies in extreme emergencies. POCUS has a sensitivity of 85% (95%CI 84–87%) and specificity of 93% (95%CI 92–95%) for pneumonia, showing it is an accurate tool for the diagnosis of pneumonia [22]. In health care settings with limited medical resources and in primary care facilities, POCUS is feasible for detecting pulmonary manifestations of malaria and sepsis [23]. In addition, POCUS has been shown to have advantages and be effective in the diagnosis and evaluation of patients in the perioperative period in the emergency departments [24]. POCUS has also been used in emergency departments for rapid assessment of the patient’s lungs and chest [25]. Therefore, POCUS is an important diagnostic tool for pneumonia due to its simplicity, accessibility, low cost, and lack of radiological hazards.

LUS had been used for the diagnosis of lung disease for a long time. Early in the COVID-19 outbreak, LUS was found to show specific findings in patients with COVID-19: irregular pleural lines on the anterior and posterior thorax bilaterally, small subpleural consolidations, white areas and thick, confluent and irregular vertical artifacts (B lines), and the presence of stripped areas bilaterally, mixed with pathologic areas. This evidence suggested that LUS could be used to diagnose and evaluate COVID-19 [26]. As COVID-19 spread across the world, the use of ultrasound became widespread and is now recommended by several national and international guidelines. For example, the WHO guidelines suggest that ultrasound can be used as a complementary alternative method for diagnostic evaluation in pregnant women and children with infection prevention and control measures [27,28]. The International Society of Ultrasound in Obstetrics and Gynecology (ISUOG) updated their guidelines stating that ultrasound can be performed to examine the fetuses and pregnant women for the diagnosis and evaluation of COVID-19 with proper protection.

### 4.2. Strengths and Limitation

A total of 14 reviews or protocols assessing the use of LUS for the diagnosis of COVID-19 have been published prior to our study, including 12 narrative reviews [29,30,31,32,33,34,35,36,37,38,39,40], one systematic review and meta-analysis [41], and one systematic review proposal [42]. The narrative reviews analyzed the role of LUS in COVID-19 from different perspectives. However, most of the reviews did not search the literature systematically or analyze the risk of bias. The conclusions of the only systematic review so far were similar to ours [41], but our search covering five databases was broader and could thus include more studies and patients, strengthening the evidence.

Our study has also some limitations. We did not include preprints because we decided to restrict the review to peer-reviewed studies to assure high quality of the meta-analysis. In addition, we did not perform subgroup analyses because the differences in patient characteristics between the studies were minor. For the scoring of LUS, we did not calculate the scores of COVID-19 because of the different scoring systems. Finally, the overall quality of the included studies was low.

## 5. Conclusions

The diagnostic agreement between LUS and CT in the diagnosis of interstitial pneumonia caused by COVID-19 is high. LUS can be therefore considered as an equally accurate alternative for CT in situations where molecular tests are not available. Particularly when performed with a point-of-care portable tool, LUS has great potential to support the diagnosis and evaluation of patients with COVID-19 in emergency or intensive care setting due to its simplicity, accessibility, low cost, and safety.

## Figures and Tables

**Table 1:** Characteristics of the included studies.

Research ID	Journals	Country/Area	Publication Date	Patients	Female, N (%)	Age (year)	Patients Enrolled Time	Setting	Sample Size	Ultrasound	CT	TP	FP	FN	TN
Şan et al. [8]	*Notfall and Rettungsmedizin*	Turkey	2 December 2020	Confirmed COVID-19	20 (50.0%)	Mean ± SD 43.8 ± 16.6	1–30 April 2020	ED	40	LUS	CT	20	3	12	5
Schmid et al. [9]	*BMC Emergency Medicine*	Germany	7 December 2020	Suspected COVID-19	NA	NA	1–25 April 2020	ED	31	LUS	CT	13	3	7	8
Karagöz et al. [10]	*Ultrasound Quarterly*	Turkey	1 December 2020	Suspected COVID-19	31 (43.0%)	Mean 51 (range 20–96)	1–15 April 2020	ED	72	BLUS	CT	32	3	1	36
Hizal et al. [11]	*Pediatr Pulmonol*	Turkey	21 October 2020	Confirmed COVID-19	NA	Children	April–May 2020	Hospital	30	LUS	CT	11	1	2	16
Chen et al. [12]	*Ultrasound Med Biol*	Spain	13 July 2020	Confirmed COVID-19	23 (45.1%)	Mean ± SD 61.4 ± 17.7	March–April 2020	ED	51	LUS	CT	37	3	0	11
Narinx et al. [13]	*Emergency Radiology*	Belgium	10 September 2020	Suspected COVID-19	49 (54.4%)	Mean ± SD 50.4 ± 16.3	28 March–20 April 2020	ED	90	POCUS	CT	22	51	0	17
Dacrema et al. [14]	*Internal and Emergency Medicine*	Italy	11 January 2021	Suspected COVID-19	32 (24.4%)	Mean ± SD 64.3 ± 14.3	21 February–15 March 2020	ED	131	LUS	HRCT	129	0	1	1
Walsh et al. [15]	*Western Journal of Emergency Medicine*	USA	28 September 2020	COVID-19	NA	≥14	4 March–19 May 2020	ED	77	LUS	CT	12	13	0	52
Lin et al. [16]	*Advanced Ultrasound in Diagnosis and Therapy*	China	6 September 2020	Asymptomatic SARS-CoV-2 infected patients	4 (44.4%)	Mean ± SD 34.0 ± 17.9	22–23 February 2020	Hospital	9	LUS	CT	2	0	1	6

LUS: Lung Ultrasound; CT: Computed Tomography; HRCT: High-resolution Computed Tomography; POCUS: Point-of-care Ultrasonography; BLUS: Bedside Lung Ultrasound; ED: Emergency Department.

## Data Availability

The datasets used during the current study are available from the corresponding author on reasonable request.

## Supplementary Materials

The following supplementary materials are available online at https://www.mdpi.com/article/10.3390/diagnostics11081351/s1.

## Abbreviations

COVID-19 = coronavirus disease 2019, LUS = Lung Ultrasound, CT = Computed Tomography, HRCT = High-resolution Computed Tomography, POCUS = Point-of-care Ultrasonography, ED = Emergency Department.
