# Supplementary material for: A Comparison of Lung Ultrasound and Computed Tomography in the Diagnosis of Patients with COVID-19: A Systematic Review and Meta-Analysis
Source: Diagnostics (Basel). 2021 Jul 27;11(8):1351. doi: 10.3390/diagnostics11081351 (PMC8394642; doi:10.3390/diagnostics11081351)
Supplement: Supplementary file 1 [file diagnostics-11-01351-s001.zip › diagnostics-1311185-supplementary.pdf]

Supplementary Material 1

Annex 1 Search strategies

PubMed

#1 "COVID-19"[Supplementary Concept]

#2 "Severe Acute Respiratory Syndrome Coronavirus 2"[Supplementary Concept]

#3 "COVID-19"[Title/Abstract]

#4 "SARS-COV-2"[Title/Abstract]

#5 "Novel coronavirus" [Title/Abstract]

#6 “2019-novel coronavirus” [Title/Abstract]

#7 “coronavirus disease-19” [Title/Abstract]

#8 “coronavirus disease 2019” [Title/Abstract]

#9 “COVID19” [Title/Abstract]

#10 “Novel CoV” [Title/Abstract]

#11 “2019-nCoV” [Title/Abstract]

#12 “2019-CoV” [Title/Abstract]

#13 OR/#1-12

#14 ultrasound*[Title/Abstract]

#15 POCUS[Title/Abstract]

#16 ultrasound [MeSH Terms]

#17 OR/#14-16

#18 "radiography, thoracic"[MeSH Terms]

#19 "computed tomography"[Title/Abstract]

#20 "radiograph*"[Title/Abstract]

#21 "imagin*"[Title/Abstract]

#22 OR/#18-#21

#23 #13 AND #17 AND #22

Cochrane library

#1 "COVID-19":ti,ab,kw

#2 "SARS-COV-2":ti,ab,kw

#3 "Novel coronavirus":ti,ab,kw

#4 "2019-novel coronavirus" :ti,ab,kw

#5 "Novel CoV" :ti,ab,kw

#6 "2019-nCoV" :ti,ab,kw

#7 "2019-CoV" :ti,ab,kw

#8 "coronavirus disease-19" :ti,ab,kw

#9 "coronavirus disease 2019" :ti,ab,kw

#10 "COVID19" :ti,ab,kw

#11 OR/#1-10

#12 MeSH descriptor: [Ultrasonography, Doppler] explode all trees

#13 (ultrasound*):ti,ab,kw

#14 (ultrasonography*):ti,ab,kw

#15 POCUS:ti,ab,kw

#16 OR/12-15

#17 #11 AND #16

Embase

#1. 'COVID-19':ab,ti

#2. 'SARS-COV-2':ab,ti

#3. 'novel coronavirus':ab,ti

#4. '2019-novel coronavirus':ab,ti

#5. 'coronavirus disease-19':ab,ti

#6. 'coronavirus disease 2019':ab,ti

#7. 'COVID19':ab,ti

#8. 'novel cov':ab,ti

#9. '2019-ncov':ab,ti

#10. '2019-cov':ab,ti

#11. 'coronavirus disease 2019'/exp

#12. OR/#1-11

#13. 'ultrasound'/exp

#14. pocus:ti,ab

#15. ultrasound:ti,ab

#16. OR/#13-15

#17. #12 AND #16

WHO COVID-19 Global literature on coronavirus disease

#1 COVID-19: ti

#2 ultrasound*: ti

#3 #1 AND #2

CBM

#1 “新型冠状病毒” [常用字段:智能]

#2 “2019-nCoV”[常用字段:智能]

#3 “2019-CoV”[常用字段:智能]

#4 “COVID-19” [常用字段:智能]

#5 "COVID 19"[常用字段:智能]

#6 “SARS-CoV-2” [常用字段:智能]

#7 “超声检查”[不加权:扩展]

#8 “超声”[常用字段:智能]

#9 “回声检查”[常用字段:智能]

#10 “回波断层摄影术”[常用字段:智能]

#11 “彩色多普勒”[常用字段:智能]

#12 “二维超声”[常用字段:智能]
